# MRI-based 3D models of the hip joint enables radiation-free computer-assisted planning of periacetabular osteotomy for treatment of hip dysplasia using deep learning for automatic segmentation

**DOI:** 10.1016/j.ejro.2020.100303

**Published:** 2020-12-18

**Authors:** Guodong Zeng, Florian Schmaranzer, Celia Degonda, Nicolas Gerber, Kate Gerber, Moritz Tannast, Jürgen Burger, Klaus A. Siebenrock, Guoyan Zheng, Till D. Lerch

**Affiliations:** a Sitem Center for Translational Medicine and Biomedical Entrepreneurship, University of Bern, Switzerland; b Department of Orthopedic Surgery, Inselspital, University of Bern, Bern, Switzerland; c Department of Diagnostic, Interventional and Paediatric Radiology, University of Bern, Inselspital, Bern, Switzerland; d Institute for Medical Robotics, School of Biomedical Engineering, Shanghai Jiao Tong University, China; e Department of Orthopaedic Surgery and Traumatology, Cantonal Hospital, University of Fribourg, Switzerland

**Keywords:** DDH, developmental dysplasia of the hip, FAI, Femoroacetabular Impingement, ROM, range of motion, FHC, femoral head center, DOC, Dice Overlap Coefficients, ASD, Average Surface Distance, GPU, Graphics processing unit, MRI, Magnetic resonance imaging, Hip dysplasia, DDH, Deep learning, Machine learning, Hip joint, Automatic segmentation

## Abstract

**Introduction:**

Both Hip Dysplasia(DDH) and Femoro-acetabular-Impingement(FAI) are complex three-dimensional hip pathologies causing hip pain and osteoarthritis in young patients. 3D-MRI-based models were used for radiation-free computer-assisted surgical planning. Automatic segmentation of MRI-based 3D-models are preferred because manual segmentation is time-consuming.

To investigate(1) the difference and(2) the correlation for femoral head coverage(FHC) between automatic MR-based and manual CT-based 3D-models and (3) feasibility of preoperative planning in symptomatic patients with hip diseases.

**Methods:**

We performed an IRB-approved comparative, retrospective study of 31 hips(26 symptomatic patients with hip dysplasia or FAI). 3D MRI sequences and CT scans of the hip were acquired. Preoperative MRI included axial-oblique T1 VIBE sequence(0.8 mm^3^ isovoxel) of the hip joint. Manual segmentation of MRI and CT scans were performed. Automatic segmentation of MRI-based 3D-models was performed using deep learning.

**Results:**

(1)The difference between automatic and manual segmentation of MRI-based 3D hip joint models was below 1 mm(proximal femur 0.2 ± 0.1 mm and acetabulum 0.3 ± 0.5 mm). Dice coefficients of the proximal femur and the acetabulum were 98 % and 97 %, respectively. (2)The correlation for total FHC was excellent and significant(r = 0.975, p < 0.001) between automatic MRI-based and manual CT-based 3D-models. Correlation for total FHC (r = 0.979, p < 0.001) between automatic and manual MR-based 3D models was excellent.

(3)Preoperative planning and simulation of periacetabular osteotomy was feasible in all patients(100 %) with hip dysplasia or acetabular retroversion.

**Conclusions:**

Automatic segmentation of MRI-based 3D-models using deep learning is as accurate as CT-based 3D-models for patients with hip diseases of childbearing age. This allows radiation-free and patient-specific preoperative simulation and surgical planning of periacetabular osteotomy for patients with DDH.

## 1 Introduction

Femoroacetabular impingement (FAI) and developmental dysplasia of the hip (DDH) are major causes of hip osteoarthritis in young and active patients [1]. DDH is an abnormality of the acetabulum, while FAI mostly affects the femoral head and neck. DDH is characterized by a static overload [2], while FAI is a painful, dynamic and early osseous conflict between proximal femur and the acetabulum which limits range of motion (ROM) [1,3,4]. Commonly used clinical tests for diagnosis have a low sensitivity and specificity [5,6]. Therefore radiological diagnosis is very important for these patients. Standard imaging assessment for hip diseases is usually based on 2D radiographs or computed tomography (CT) scans. However, 2D radiographs are not specific and cannot visualize the exact location of the deformity [7].

In contrast, previous studies showed, that CT-based 3D-models allow exact surgical planning [8,9] of hip arthroscopy [[10], [11], [12], [13]] for FAI or periacetabular osteotomy [2] for DDH. But CT scans should not be used for young patients due to the radiation exposure [14], especially in patients of child-bearing age. Recently, computer-assisted 3D-MRI-based diagnosis of DDH and FAI was introduced to overcome these problems [15]. For these patients, MRI-based osseous 3D-models [16] of the hip joint represent a radiation-free method that can provide a circumferential analysis of the deformity and calculation of femoral head coverage. But these MRI-based 3D-models were obtained by manual segmentation, and this is a very time-consuming process (up to 3−4 hours), not applicable for clinical routine. Therefore automatic segmentation was investigated.

Femoral head coverage is an important parameter for treatment of patients with DDH [17]. The main objective of corrective surgery is to increase femoral head coverage to optimize the orientation of the weight-bearing zone [17]. This can reduce the joint contact pressure and therefore reduce the risk for premature development of osteoarthritis of the hip joint [18,19]. Previous methods for calculation of the femoral head coverage used 2D pelvic radiographs [20] or cumbersome and complex assumptions [21]. A CT-based method for calculation of the femoral head coverage was recently applied for patients with DDH [22]. But segmentation of CT-based 3D models has considerable radiation exposure [14].

Previous studies [[23], [24], [25]] investigated automatic segmentation of 3D-model from hip MRI and used deep learning for detection of hip fractures [26]. But, they performed 3D segmentation for the proximal femur only, while the segmentation of acetabulum was not performed [23,24]. Furthermore, only few studies evaluated the segmented 3D models in clinical routine for symptomatic patients. To the best of our knowledge, this is one of the first studies that used a radiation-free, patient-specific and non-invasive method for preoperative planning using automatic segmentation of MR-based 3D-models based on deep learning.

The purposes of this study were (1) to investigate the difference and the (2) correlation for femoral head coverage and other parameters between automatic segmentation of MR-based and of CT-based 3D-models and between automatic and manual segmentation of MRI-based 3D models (3) to test feasibility of simulation and planning of periacetabular osteotomy using MRI-based 3D models of symptomatic patients with DDH and FAI.

## 2 Patients and methods

### 2.1 Patients

Following IRB-approval we performed a comparative, retrospective study of a series of 31 hips from 26 symptomatic patients with FAI or DDH who presented at our university centre for hip preservation between 03/2016 and 02/2017. Patients were referred to imaging based on a history of hip pain, clinical and radiographic findings consistent with hip impingement or hip instability. We performed automatic segmentation of MR-based osseous 3D-models and compared them to manual segmentation of MR-based and CT-based models, of the hip joint of the same patients.

The inclusion criteria of data are as follows: availability of standard anteroposterior radiographs, availability of both standardized CT scan and a direct MR arthrography of the same hip including the entire pelvis, radiographic signs of skeletal maturity and the presence of hip pain at the time of image acquisition. The institutional imaging database was reviewed for all patients in which a CT scan and direct MR arthrography of the pelvis were performed between 03/2016 and 02/2017. Finally, 31 hips of skeletal mature patients with MR and CT scans remained in the study group.

All patients were evaluated for hip preservation surgery in the outpatient clinic from the author’s institution by experienced surgeons(MT, KAS). During routine clinical evaluation the patient history was acquired the hip ROM was measured, and the anterior and posterior impingement tests [3] were evaluated. Routinely we obtained anteroposterior pelvic radiographs in a standardized manner [3] and MR arthrography of the hip for the diagnostic preoperative evaluation for hip-preserving surgery.

Mean age was 27 ± 7 years and 52 % were women (Table 1). Of the included 31 hips, 7 hips(23 %) had a cam deformity [27], 5 hips(16 %) had a pincer deformity (Table 1). Five hips(16 %) had cam deformity combined with decreased femoral version [28] while 7 hips(23 %) had increased femoral version [29,30]. The definition of cam and pincer-type deformities was in accordance with previously published criteria on conventional anteroposterior pelvic radiographs [31] shown in Table 2.

**Table 1:** Demographic and radiological data of the study group are shown. Values are expressed as mean ± SD and range in parenthesis unless otherwise indicated.

Parameter	Total
Hips (patients)	31 (26)
Age (years)	27 ± 7 (17–41)
Sex (% men)	48
Side (% right)	61
Bilateral hip (%)	16
Height (cm)	176 ± 6 (163–186)
Weight (kg)	83 ± 20 (49–117)
BMI (kg/m^2^)	27 ± 6 (18–38)
LCE angle (°)	31 ± 10 (12–56)
Acetabular index (°)	4 ± 8 (-15 – 20)
Extrusion index (%)	20 ± 8 (1–36)
Alpha angle (°)	51 ± 11 (35–84)
Femoral torsion (°)	25 ± 12 (7–54)
Acetabular version (°)	18 ± 6 (7–31)
McKibbin Index (°)	43 ± 15 (20–75)
cam-type deformity	7 hips (23 %)
pincer-type deformity	5 hips (16 %)
Mixed-type deformity	3 hips (10 %)

**Table 2:** Definitions for hip deformities are shown below.

Deformity	Definition
Cam deformity	Alpha-angle [32] exceeding 60° [33]
Pincer-type-FAI [34,35,36]	LCE-angle exceeding 40° [31]
Mixed-type FAI	combination of cam and pincer-type-FAI [37]
DDH	LCE-angle<22° [31]
Increased femoral version	Femoral version (>25°) [29]
Decreased femoral version	femoral version(<10°)

DDH = developmental dysplasia of the hip; FAI = Femoroacetabular impingement. Normal values for femoral version was 10−25° [38].

### 2.2 Imaging technique

**Fig. 1:**
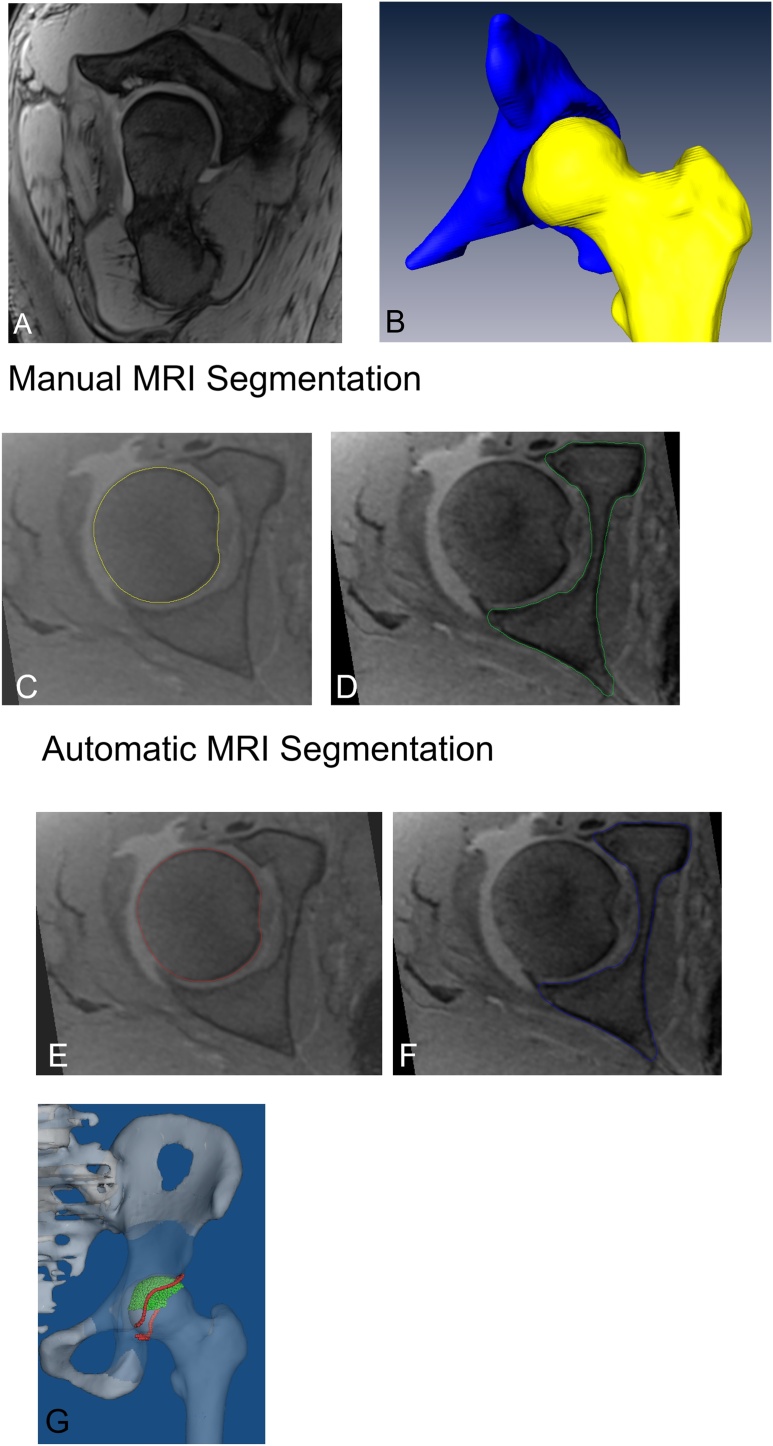
**A-G** The MRI image (A), an MRI-based 3D model (B) and comparison between manual (C and D) and automatic MRI-based 3D model (E and F) of the femur (C and E) and of the acetabulum (D and F) are shown. Calculation of femoral head coverage (G) is shown.

We used a standardized protocol for MR arthrography on 3 T scanner (Siemens Medical Solutions, Erlangen, Germany) with large flexible surface coils and multiplanar PD-w images in coronal, sagittal, axial and radial orientation [39,40]. In addition, we used an unilateral high-resolution 3D sequence for reconstruction of 3D-models of the hip (Fig. 1A). The unilateral 3D sequence of the hip had a field of view(FOV) including the hip joint, with the unilateral acetabulum including the ischial tuberosity and the proximal femur including the greater trochanter (Fig. 1A): axial-oblique 3D volumetric interpolated breath-hold examination (VIBE, Fig. 1A) was used for the affected unilateral hip joint (repetition time/echo time, 15/3.3 ms, flip angles of 4° and 24°, slice thickness of 0.8 mm, 160 × 160 mm field of view, a matrix size of 192 × 192, isotropic voxel size of 0.8 mm3, acquisition time of 9 min for 128 slices.

The CT scans were acquired with a dual source scanner (DSCT) or 128-slice multi-detector (Somatom Definition Flash/Edge, Siemens Medical Solutions, Erlangen, Germany) in accordance with previous reports [28]. The scanned volume covered the complete pelvis and a second volume covering the distal femoral condyles. Scan parameters were: collimation 128 × 0.6 mm, voltage 100/120 kVp; pitch 0.8. Automated-attenuation based tube current modulation was used (40 mA reference). One mm thick images were reformatted (convolution kernel I31f). The mean Dose-length product (mGy × cm) of the patient series was 295 ± 124 with a range of 138–713.

### 2.3 Manual segmentation of 3D models

Segmentation of CT-based and MR-based osseous 3D-models for each hip joint were performed manually, as previously described [58]. Each CT-based or MR-based 3D surface model included a 3D surface model of the acetabulum and the proximal femur. The manual segmentation of 3D-model was performed semi-automatically for each hip joint using a threshold-based method by commercial software Amira Visualization Toolkit (Visage Imaging Inc, Carlsbad, CA, USA) by two observers(TDL and CD, Fig. 1B). Segmentation of 3D models based on CT scans, was performed on axial CT scans with a slice thickness of 1 mm and took 90−120 min for each hip joint. Segmentation of MR-based 3D models (Fig. 1C and D) was performed on 1 mm thick reformatted true axial images from the 3D axial-oblique T1 VIBE images and took 3−4 hours.

### 2.4 Automatic segmentation

**Fig. 2:**
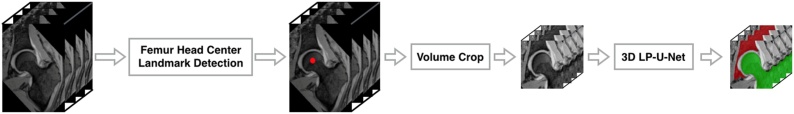
A schematic illustration of the two-stage deep learning based method for fully automatic hip MRI joint segmentation. The femoral head center is detected by the landmark detection network, and then we crop the hip joint data around the femoral head center. Finally the LP-U-Net is applied to segment the cropped hip joint.

**Fig. 3:**
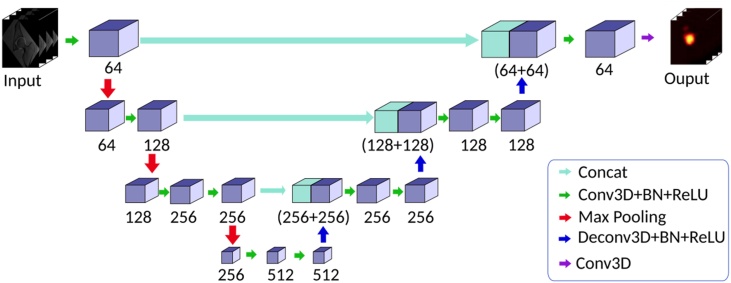
A schematic view of the landmark detection network for femoral head center. The neural network adopts an encoder-decoder architecture with skip connections. The encoder takes MRI data as input and generates high-dimensional feature vector, while the decoder takes the high dimensional feature vector as input and generates the landmark heatmap. The number below each block is the number of feature stack.

Automatic segmentation of MRI-based 3D-models (Fig. 1E and F) was performed using deep-learning. We developed a deep-learning-based fully automatic method for 3D hip joint segmentation from MR images. Deep learning is a part of the big family of machine learning and is based on artificial neural networks, especially on Convolutional Neural Networks (CNN). The used method (Fig. 2) for fully automatic hip joint segmentation of MRI images consisted of two stages: First, the femoral head center (FHC) was detected by a landmark detection network (Fig. 3). The landmark detection network was a fully CNN, which can directly map a whole volumetric data to its volume-wise heatmap. And the location of highest value in the heatmap was recognized as the detected landmark. The detected FHC allowed us to crop the original data including the joint space, femoral head and the acetabulum. Second, another neural network was trained to segment the cropped hip joint data. The hip joint segmentation network was based on the LP-U-net which was introduced in a previous study [59], in which holistic decomposition convolution and dense upsampling convolution were applied at the beginning and end of the 3D-U-net, respectively. LP-U-net has one essential advantage: the reduction of the GPU memory for sub-sequential processing while incorporating larger context information for a better performance. In order to avoid overfitting of our deep learning model, we used several techniques to improve the generalization of our model (Supplemental material Fig. 1 and 2 with loss curves of training and testing on three groups). We did not perform hyperparameter tuning on the 3 groups for hip joint MR segmentation and landmark detection, and all hyperparameters were obtained in our previous work on based on another hip MR dataset [28] (Supplemental material with detailed information).

We conducted a standard 3-fold cross validation study using the 3D axial-oblique T1 VIBE MR images of the unilateral hip joint of the 31 hips. Specifically, we randomly split the 31 data into 3 groups. Each time, one group was taken as testing data, and the rest of two groups were used as training data. We repeated this process three times such that each group was used once as testing data. We controlled that the hips of the same patients are not present in both training and testing dataset. This data splitting strategy of 3-fold cross-validation allowed us to test our algorithm three times in blind testing on unknown data. We used Dice Overlap Coefficients(DOC) and Average Surface Distance(ASD) to evaluate accuracy (main evaluation metrics). Our method was implemented with Python using TensorFlow framework on a workstation with a 3.6 GHz Intel® i7 CPU and a GTX 1080 Ti graphics card with 11 GB GPU memory.

To answer the first question, we used commercial software for 3D reconstruction(AMIRA) to calculate accuracy. Automatic and manual MRI-based models of the same hip joint were compared in this software (Fig. 1C and D). Transform editor from AMIRA was used to align two surfaces, and then the surface distance error between two 3D models was calculated. The manual MRI-based 3D model served as gold standard. We used DOC and ASD as the outcome parameters.

To answer the second and third question, we used a specific software to calculate six diagnostic parameters including anterior, posterior and total femoral head coverage (Fig. 1G), anteversion, inclination and the extrusion index [8,9]. This software was developed for planning of periacetabular osteotomy. This software was based on a validated medical research framework [41] and was described in detail in previous publications [41,42].

We used Winstat software(R. Fitch Software, Bad Krozingen, Germany) to perform statistical analysis. Normal distribution was tested using the Kolmogorov-Smirnov test for continuous variables. Pearson’ correlation coefficient was used because the variables were normally distributed. Absolute mean differences were calculated for continuous variables. Interobserver correlation coefficient was calculated using Medcalc software (Version 17.6;MedCalc Software, Ostend, Belgium). Bland Altman analysis was performed to search for a systematic error. Intraclass correlation coefficient(ICC) was performed for comparing the two methods.

## 3 Results

(1)The dice coefficient between automatic and manual segmentation of MRI-based 3D-models was 97 ± 2% for the acetabulum and 98 ± 1% for the femur (Table 3). The mean surface difference between automatic and manual segmentation of MRI-based 3D-models were 0.3 ± 0.5 mm for the acetabulum and 0.2 ± 0.1 mm for the proximal femur (Table 3), respectively. The detailed results of the automatic segmentation can be found in the supplemental material.

**Table 3:** Accuracy of the automatic segmentation of MRI-based 3D models by our proposed 3D LP-U-net compared to manual segmentation of MRI-based 3D models serving as gold standard is shown.

Parameters	Acetabular models	Femoral models
Number of hips	31	31
Dice coefficient (%)	97 ± 2 (92–99)	98 ± 1 (93–99)
Precision (%)	96 ± 3 (89–99)	98 ± 2 (92–100)
Recall (%)	97 ± 2 (89–100)	97 ± 3 (87–100)
Mean surface distance (mm)	0.3 ± 0.5 (0.1–3)	0.2 ± 0.1 (0.1 – 0.5)
Maximum (mm, Hausdorff distance)	9.7 ± 8 (3–39)	5.7 ± 2 (2–13)

**Fig. 4:**
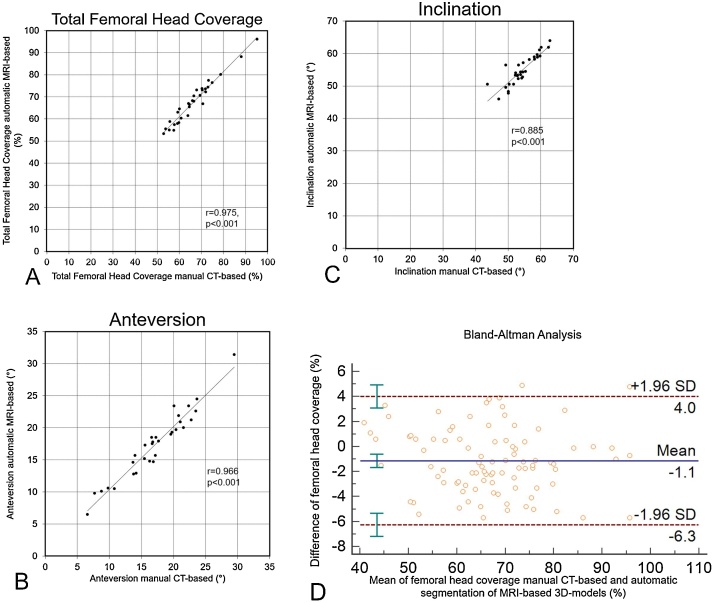
**A-D** Correlation for total femoral head coverage(A), for anteversion (B) and for inclination (C) between automatic MRI-based and CT-based 3D models are shown. Bland-Altman analysis of the total femoral head coverage showed a mean difference of 1.1 % (D).

Values are expressed as mean ± SD and range in parenthesis unless otherwise indicated.

(2)Correlation for total femoral head coverage (r = 0.975, p < 0.001) between CT and automatic MR-based 3D-models was excellent and significant (Fig. 4A). Correlation for anteversion and inclination(r = 0.966 and r = 0.885, p < 0.001) was excellent and significant (Fig. 4B and C). The mean absolute difference for inclination between manual CT-based (54°±4° [range, 44°–63°]) and automatic MR-based (55°±5° [range, 46°–64°]) 3D-models was 1°±2° (0°–7°, Table 4). The mean absolute difference for anteversion was 1°±1° (range, 0°–3°) and for total femoral head coverage was 2%±1% (range, 0%–5%,Table 4). The Bland-Altman analysis for total femoral head coverage between CT and automatic MR-based 3D models showed no systematic error (Fig. 4D). The ICC for total femoral head coverage between CT and automatic MR-based 3D models was 0.97 (range 0.96 to 0.98).

**Table 4:** Results of the automatic MRI-based and CT-based calculation of diagnostic parameters using specific software are shown.

Parameters	CT-based 3D models	Automatic MRI-based 3D models	Difference CT vs MRI	Absolute Difference CT vs MRI
Number of hips	31	31		
Inclination (°)	54 ± 4 (44–63)	55 ± 5 (46–64)	−1 ± 2 (-7 – 2)	1 ± 2 (0–7)
Anteversion (°)	17 ± 5 (7–30)	17 ± 5 (7–31)	0 ± 1 (−3 – 2)	1 ± 1 (0–3)
LCE angle (°)	28 ± 10 (10–50)	28 ± 10 (9–46)	1 ± 2 (−4 – 7)	2 ± 2 (0–7)
Extrusion index (%)	21 ± 8 (1–37)	19 ± 9 (0–36)	2 ± 2 (−5 – 6)	3 ± 2 (0–6)
Total femoral head coverage (%)	67 ± 9 (53–95)	68 ± 10 (53–96)	−1 ± 2 (-5 – 4)	2 ± 1 (0–5)
Anterior femoral head coverage (%)	59 ± 13 (42–98)	60 ± 13 (40–93)	−1 ± 3 (−6 – 5)	3 ± 2 (0–6)
Posterior femoral head coverage (%)	74 ± 9 (56–93)	75 ± 10 (58–99)	−1 ± 3 (−6 – 5)	2 ± 2 (0–6)

Values are expressed as mean ± SD and range in parenthesis unless otherwise indicated.

**Fig. 5:**
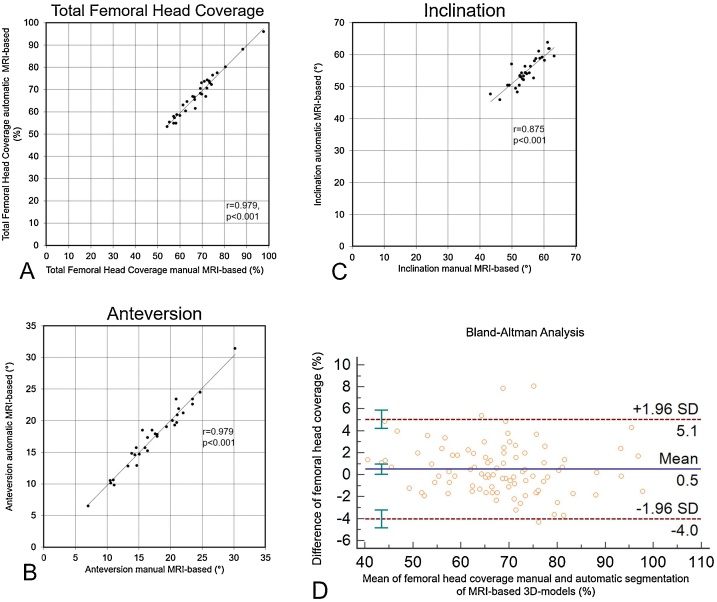
**A-D** Correlation for total femoral head coverage (A), anteversion (B) and inclination (C) between automatic and manual MRI-based 3D models are shown. Bland-Altman analysis of the femoral head coverage showed a difference of 0.5 % (D).

Correlation for total femoral head coverage (r = 0.979, p < 0.001) between automatic and manual MR-based 3D models was excellent and significant (Fig. 5A). Correlation for anteversion and inclination (r = 0.979 and r = 0.875, p < 0.001) was excellent and significant (Fig. 5B and C). The mean absolute difference for inclination between manual (55°±5° [range, 43°–63°]) and automatic MR-based (55°±5° [range, 46°–64°]) 3D models was 2°±2° (0°–7°, Table 5). The mean absolute difference for anteversion was 1°±1° (range, 0°–3°) and for total femoral head coverage was 2%±1% (range, 0%–5%, Table 5). The Bland-Altman analysis for total femoral head coverage between automatic and manual MR-based 3D models showed no systematic error (Fig. 5D). The ICC for total femoral head coverage between automatic and manual MR-based 3D models was 0.98 (range 0.97 to 0.99).

**Table 5:** Results of the manual and automatic MRI-based 3D models used for calculation of diagnostic parameters using specific software are shown.

Parameters	Manual MRI-based 3D models	Automatic MRI-based 3D models	Difference manual vs automatic	Absolute Difference manual vs automatic
Number of hips	31	31		
Inclination (°)	55 ± 5 (43–63)	55 ± 5 (46–64)	0 ± 2 (−7 – 4)	2 ± 2 (0–7)
Anteversion (°)	17 ± 5 (7–30)	17 ± 5 (7–31)	0 ± 1 (−3 – 2)	1 ± 1 (0–3)
LCE angle (°)	28 ± 10 (10–53)	28 ± 10 (9–46)	1 ± 2 (−3 – 6)	2 ± 2 (0–6)
Extrusion index (%)	19 ± 8 (1–35)	19 ± 9 (0–36)	1 ± 2 (−5 – 4)	2 ± 1 (0–5)
Total femoral head coverage (%)	68 ± 10 (54–98)	68 ± 10 (53–96)	1 ± 2 (−3 – 5)	2 ± 1 (0–5)
Anterior femoral head coverage (%)	61 ± 13 (41–98)	60 ± 13 (40–93)	1 ± 2 (−2 – 8)	2 ± 2 (0–8)
Posterior femoral head coverage (%)	75 ± 9 (56–97)	75 ± 10 (58–99)	0 ± 3 (−4 – 8)	2 ± 2 (0–8)

Values are expressed as mean ± SD and range in parenthesis unless otherwise indicated.

**Fig. 6:**
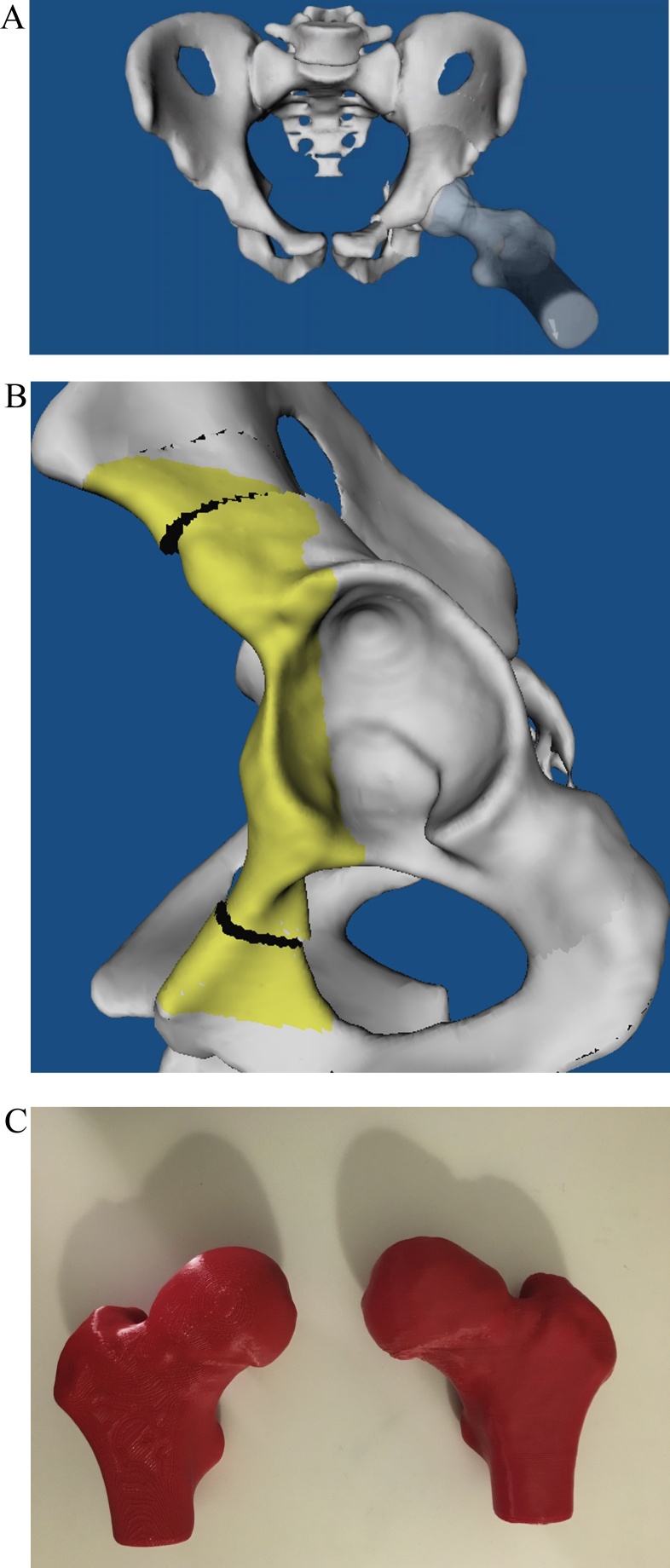
**A-C**. An AP view of MRI-based 3D model of the pelvis with a simulated periacetabular osteotomy (PAO) on the left side is shown (A). Lateral view of the same pelvis is shown (B). 3D Printed plastic models of MRI-based 3D models of the bilateral proximal femur of the same patient are shown (C).

(3) Feasibility of simulation and planning of periacetabular osteotomy using MRI-based 3D-models was possible for all hips (100 %) with hip dysplasia or acetabular retroversion(Fig. 6 and Video 1). 3D Printing of MRI-based 3D-models was feasible (Fig. 6D).

## 4 Discussion

The aim of this study was to investigate the accuracy of automatic segmentation of MRI-based 3D-models, and the correlation for femoral head coverage and other outcome parameters comparing manual and automatic segmentation of 3D models using MRI. Most importantly, an excellent correlation for femoral head coverage (Fig. 4A), anteversion (Fig. 4B) and inclination (Fig. 4C) between manual segmentation of CT-based and automatic segmentation of MRI-based 3D-models (Fig. 1E and F) was found. For FAI and DDH, femoral head coverage and anteversion are important diagnostic parameters for the decision making for surgical therapy in hips with pincer impingement [43] and can be used for surgical planning [8,9] of periacetabular osteotomy or hip arthroscopy [13,44]. Accuracy in terms of ASD was below 1 mm between manual and automatic MRI-based 3D-models (Table 3). This is one of the first studies that used a radiation-free and patient-specific method for automatic segmentation of 3D-models.

The accuracy of our results for segmentation of MRI-based 3D-models (Table 3) is comparable with the published results in the literature. The 3D U-Net [45] for automatic medical image segmentation is one of the state-of-the-art methods [40]. Previous studies used the 3D U-Net [24,25] for segmentation of MRI-based 3D-models of the hip. The accuracy of the results from 3D U-Net on our dataset was slightly lower than the results from the current 3D LP-U-net (Fig. 2, Fig. 3). Specifically, 3D U-Net achieved a DOC of 95 % and 97 % for acetabulum and femur [24,25], while a DOC of 97 % and 98 % (Table 3) was achieved in the current study. In addition, an ASD of 0.5 mm and 0.4 mm was described for the 3D U-Net, but in the current study an ASD of 0.3 mm and 0.2 mm was achieved (Table 3), respectively.

In addition, automatic 3D segmentation methods from CT were introduced in previous works [46,47]. Others proposed 3D feature-enhanced network for femur segmentation from CT images with a DOC of 96.8 % [48]. In another study, a multi-atlas segmentation constrained Graph method(MASCG) was proposed and they reported an ASD of 0.3 mm for the pelvis and the proximal femur [46], this is a comparable ASD compared to our study (Table 3). But all these methods were performed on CT images [48], and only few studies investigated automatic segmentation using MR images [23,24]. These studies investigated automatic 3D segmentation of the proximal femur based on 3D-MR images using deep learning [23,24]. However, these studies only segmented the femur, and the segmentation of acetabulum was not performed.

To the best of the authors’ knowledge, we found no other study comparing automatic segmentation of MRI-based models with CT-based 3D-models of the hip joint of symptomatic patients. Some previous studies compared the segmentation of 3D-models in cadavers [49,50] or animal models [50] or with various methods for segmentation [15]. A recent study used 3D-MRI for the evaluation of acetabular labrum tears [51]. Some other studies explored the segmentation of MRI-based 3D-models with different MRI protocols, but they are difficult to use in clinical routine [16,52]. This could be due to the small FOV, longer acquisition time, different bone intensity and unclear boundaries between bone and soft tissues.

Comparing the results of the six evaluated diagnostic parameters, most of the previous studies used CT-based 3D-models. Their published results [46,47] are in line with the results we found in this study(Table 4). The mean difference [47] of the diagnostic parameters are in accordance with other studies. Another study compared 3D-models based on fully automatic CT segmentation(FACTS) with 3D-models based on manual CT segmentation [47]. They reported a difference of 2.0 ± 1.5°, 2.1 ± 1.6° and 3.5 ± 2.3 % for anteversion, inclination and femoral head coverage, respectively [47]. Comparing manual and automatic MRI-based 3D models, we reported a mean difference of 1 ± 1°, 2 ± 2°, and 2 ± 1% for anteversion, inclination and total femoral head coverage (Table 5), respectively.

This study investigating the automatic segmentation of MRI-based 3D hip joint models has important implications. To overcome the mentioned problems of 2D imaging, 3D-imaging is preferred as they can provide more diagnostic information and allow patient-specific surgical planning. CT scan is mostly common used for 3D-imaging for the diagnosis of FAI and DDH. Furthermore, CT arthrography has demonstrated to have the strongest overall diagnostic accuracy [6] in a recent systematic review including 25 studies. However, CT scans are not frequently performed in our institution because of radiation exposure in this typically young patient group. In addition, recently a 4D-CT method for the diagnosis of FAI was proposed, but it used three times the dose of a routine CT examination of the pelvis [53]. Recently, manual MRI-based segmentation for osseous 3D-models were investigated for surgical planning [58]. But manual MRI segmentation is very time-consuming. It took 3−4 hours to manually reconstruct a 3D-model based on MR scans while the deep learning method only took 1−2 min. The method used in the current study showed a fast and accurate automatic segmentation. The used method for simulation of periacetabular osteotomy could be further used for surgical navigation using MRI-based 3D models. In addition, this could be used for 3D printing based on MRI-based 3D-models. 3D Printing is a novel tool for preoperative planning of cam resection [54,55] and can influence the location of the planned osteoplasty [56].

This study has the following limitations. First, no clinical followup of the patients was performed. Second, only hips without osteoarthritis (Tönnis grade<2), without previous operations, and with the complete MRI and CT were included. Some hips were excluded because MR images had severe artifacts when patients had previous operations and screw fixation. This limits the use of our proposed method for automatic 3D-model segmentation for patients with implants. Additionally, the used MRI sequence was originally used for cartilage analysis and was not used routinely. Third, a low number of hips with protrusio acetabuli(2 hips[6%], Table 1) were available. But this may be also a strength, because it demonstrated that our proposed method also works for these hips with uncommon deformities with a low prevalence. Future studies could investigate automatic segmentation of MR-based models on a larger dataset with more complex deformities (e.g. posttraumatic deformities). Last, only skeletal mature patients were included and thus cannot extrapolate our findings to patients with pediatric hip disease.

To overcome the mentioned problems of radiographs and CT scans, automatic segmentation of MRI-based 3D-models was used with the aim to replace manual segmentation of CT-based 3D-models. And the proposed deep learning based method for automatic hip joint 3D-reconstruction showed promising results. Based on the results of this study, it is possible to reduce preoperative CT scans. This could reduce the lifetime risk of malignancy and the radiation dose of a pelvic CT scan ranging from 2.9 to 5 mSv [14]. This is especially beneficial for patients of childbearing age with hip pain due to FAI or DDH, and pediatric patients with SCFE [57].

## 5 Conclusion

Automatic segmentation of MRI-based 3D-models of the hip joint based on deep learning showed promising results with an average surface difference below 0.5 mm for both acetabulum and femur. More importantly, the correlation for six diagnostic parameters was excellent when comparing automatic with manual segmentation of MR-based 3D-models. Based on these results, it is possible to use automatic segmentation of MR-based 3D-models in the future. In addition, this allows radiation-free and patient-specific preoperative surgical planning of periacetabular osteotomy, and could be beneficial for patients of childbearing age with hip pain due to FAI or DDH.

## Funding statement

Three authors have received funding from the Swiss National Science Foundation (MT, FS, TDL). Each author certifies that his or her institution approved the human protocol for this investigation, that all investigations were conducted in conformity with ethical principles of research, and that informed consent for participation in the study was obtained.

## CRediT authorship contribution statement

**Guodong Zeng:** Data curation, Formal analysis, Software. **Florian Schmaranzer:** Writing - original draft. **Celia Degonda:** . **Nicolas Gerber:** Project administration. **Kate Gerber:** Project administration. **Moritz Tannast:** Funding acquisition, Supervision, Writing - review & editing. **Jürgen Burger:** Supervision. **Klaus A. Siebenrock:** Funding acquisition, Supervision, Writing - review & editing. **Guoyan Zheng:** Software. **Till D. Lerch:** Data curation, Formal analysis, Writing - original draft.

## Declaration of Competing Interest

The authors declare that they have no known competing financial interests or personal relationships that could have appeared to influence the work reported in this paper.
