# Supplementary material for: MRI-based 3D models of the hip joint enables radiation-free computer-assisted planning of periacetabular osteotomy for treatment of hip dysplasia using deep learning for automatic segmentation
Source: Eur J Radiol Open. 2020 Dec 18;8:100303. doi: 10.1016/j.ejro.2020.100303 (PMC7753932; doi:10.1016/j.ejro.2020.100303)
Supplement: Supplementary file 1 [file mmc1.docx]

**Supplemental material for description of the automatic segmentation using deep learning**

**Overfitting**

In order to avoid overfitting of our deep learning model, this paper uses several techniques to improve the generalization of our model on unseen test data. In particular, we used L2 norm weight regularization, where the square root of the sum of the squared training parameter values was calculated as penalty. This weight regularization was used for all convolution layers in our neural network. In this way, all learned parameters were encouraged to have small weights, which resulted in a lower capacity of the model and reduced the probability of over-fitting the model on the training dataset. In addition, we have also tried to train our neural network with more samples to reduce the over fitting. This so called data augmentation (technical term) was used to enlarge our training samples. In particular, all 3D MRI data were augmented by rotating them 90, 180, and 270 degrees around the z-axis and flipped horizontally around the y-axis. Before each sub-volume was fed into the neural network for training, it was additionally augmented with a random Gaussian noise from a normal distribution N (0, 0.05). The loss curves of training and testing on three groups are shown in Fig.1 and Fig.2. The average testing results from 3 groups are shown in Table 2, which reports an average Dice of 97% and 98% for acetabular and femoral models. This test result showed that our algorithm is not overfitting during training and that it achieves good segmentation results on unseen test data.


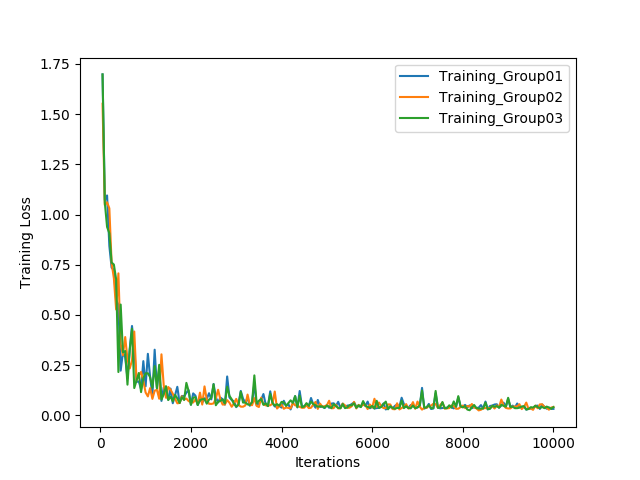


Figure 1. The training loss curve of the three groups is shown.


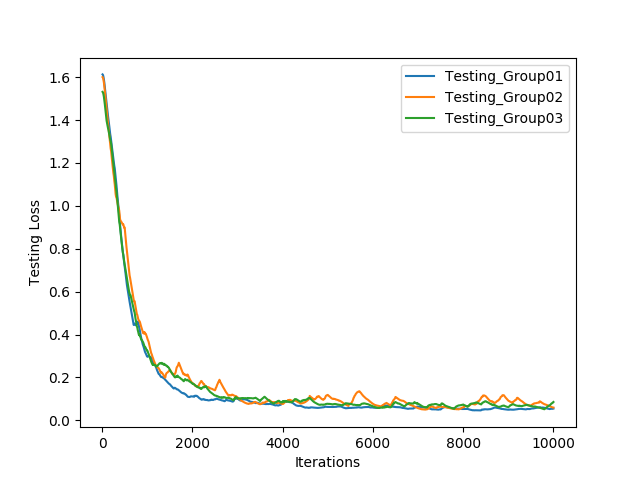


Figure 2. The testing loss curve of the three groups is shown

**Data splitting**

We performed a 3-fold cross-validation on the 31 3D axial-oblique T1 VIBE MR images of the unilateral hip joint from 26 patients. In each group, 20 data were used as training and then blindly tested on the unseen holdout 10 or 11 data. We make sure that the hip joint MRIs from the same patient are not present in both training and testing dataset. This data splitting strategy of 3-fold cross-validation allows us to blindly test our algorithms three times on unseen holdout data.

**Parameter finetuning**

We did not perform hyperparameter tuning on the 3 groups training of hip joint MR segmentation and landmark detection, and all hyperparameters were obtained in our previous work on segmenting another hip MR dataset [58]. The reason for this is that these hyperparameters are not designed for a specific task and they have already been successfully used for different tasks (e.g. proximal femoral segmentation [24,25], cartilage segmentation [59], pancreas segmentation and intervertebral disc segmentation [61]). Specifically, all parameters were trained from scratch and initialized from a Gaussian distribution (μ = 0, σ = 0.01). All parameters were updated by the algorithm of stochastic gradient decay (SGD) with momentum (beta = 0.9, weight decay = 0.005). Each neural network was trained in 10,000 iterations. The initial learning rate was 0.001 and halved every 3000 iterations.

**Landmark detection network**

Landmark detection is achieved from a fully convolutional network, which takes the hip MRI as input and outputs a heatmap regression. The ground truth heatmap of the landmark is generated by applying a Gaussian distribution N (0, 1) at the landmark position. The maximum value is 1.0 and is set at the landmark position, while the minimum value is 0.0. Further the distance to the landmark position, the lower is the heatmap value. In the testing phase, each landmark coordinate is obtained by the position of maximum value of the predicted heatmap.

The architecture of the proposed neural network for landmark heatmap regression is shown in Fig. 3. It is an encoder-decoder structure that takes the input MRI as input and outputs a regressive heatmap prediction. The encoder is a single contracting path designed for feature learning at different levels and consists of multiple convolutional and max-pooling layers. The decoder is an expanding path used to enable heatmap regression using convolution layers. It is a fully convolutional neural network and can accept images of any size as input. All activation outputs before the max pooling layers in the contracting path are connected to symmetrical layers of each expanding path. These multiple skip connections are used to restore the context lost during down-sampling operations. The proposed heatmap regression neural network is optimized by a mean squared error loss between the predicted heatmaps and the corresponding ground-truth heatmaps. We conducted a standard 3-fold cross-validation for the landmark detection of femur head center. The detection for the landmark of FHC reported an average error distance of 3.2 mm, which is accurate enough to crop the hip joint region from the input MRI for the subsequent segmentation task in the second stage.

**MRI segmentation network**

We have developed an automatic segmentation method for MRI-based hip joints using deep learning. Deep learning is a part of machine learning and is mainly driven by convolutional neural networks (CNN) which can automatically learn different levels of features directly from the raw input data. The method for segmentation of the hip joint developed in this paper is built on LP-U-net [61]. In the LP-U-net, holistic decomposition convolution (HDC) and dense upsampling convolution (DUC) were applied at the beginning and the end of the 3D-U-net, respectively. HDC consists of a periodic down-shuffling operation with shuffle factors of (n_x_, n_y_, n_z_) and a normal convolutions, while DUC consists of a periodic up-shuffling operation with shuffle factors of (n_x_, n_y_, n_z_) and a normal convolutions. With the together use of HDC and DUC, LP-U-net has the advantage of significantly reducing the GPU memory for sub-sequential processing while incorporating larger context information for a better performance. LP-U-net was trained with the Dice loss proposed in V-net[60].


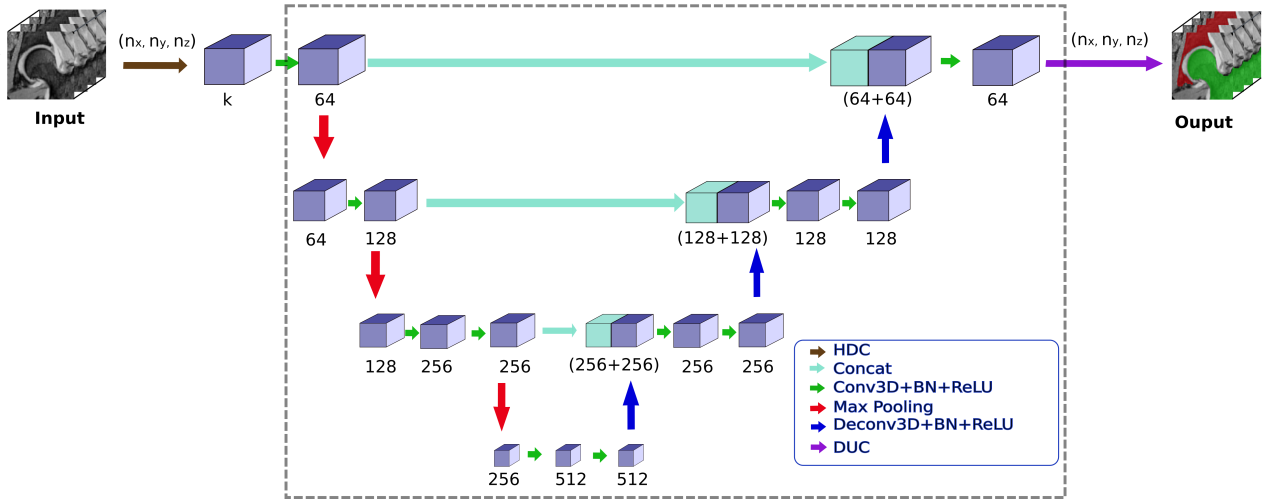


Figure 3. The hip joint segmentation network based on the LP-U-net is shown here.

**Evaluation metrics**

Assuming the automatically segmented set of voxels as AS and the manually defined ground truth as GT, the metrics of DOC, ASD and HD are defined as below:

(1) Dice Overlap Coefficients (DOC) quantifies the similarity between AS and GT:


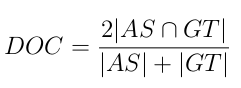


where the operator |·| returns the number of voxels contained in a set.

(2) Average Surface Distance (ASD)

We first define a distance measure for a voxel x to a set of voxels A as: d(x, A) = min { d(x, y) | y∈A } , where d(x, y) is the Euclidean distance of the voxels incorporating the real spatial resolution of the volume data. Average Surface Distance (ASD) is defined as the average of all the distances from points on the boundary of AS (we denote them as B_AS_ ) to the boundary of GT (B _GT_ ):


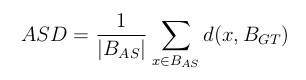


(3) Hausdorff Distance (HD)

We further define the directed Hausdorff measure from a set A to a set B as the maximum distance for all points in A to the closest point in B. Based on this, Hausdorff Distance (HD) is defined as as the maximum distance between two objects: Hausdorff measure from a point set A to a point set B or from Hausdorff measure from set B to set A.

**Full report of the results for each test set**

A full report of the results for each test set of 3-fold cross validation are shown below:

Table 1: Accuracy of the automatic segmentation of MRI-based 3D models on Group01 by our proposed 3D LP-U-net compared to manual segmentation of MRI-based 3D models serving as gold standard is shown.

| Metrics | Acetabular models | Femoral models |
| --- | --- | --- |
| Dice Overlap Coefficients (%) | 97.3±0.7 (96.2–98.4) | 97.9±0.5 (97.5–98.7) |
| Average Surface Distance (mm) | 0.19±0.05 (0.11-0.28) | 0.19±0.04 (0.11-0.24) |
| Hausdorff Distance (mm) | 7.6±3.39 (4.7-14.1) | 5.85±2.15 (4.02-10.1) |

Table 2: Accuracy of the automatic segmentation of MRI-based 3D models on Group02 by our proposed 3D LP-U-net compared to manual segmentation of MRI-based 3D models serving as gold standard is shown.

| Metrics | Acetabular models | Femoral models |
| --- | --- | --- |
| Dice Overlap Coefficients (%) | 96.8±1.5 (93.7–98.5) | 97.1±1.6 (92.8–98.6) |
| Average Surface Distance (mm) | 0.22±0.13 (0.08-0.47) | 0.25±0.13 (0.12-0.49) |
| Hausdorff Distance (mm) | 9.41±7.32 (3.4-29.7) | 6.79±2.62 (3.83-12.5) |

Table 3: Accuracy of the automatic segmentation of MRI-based 3D models on Group03 by our proposed 3D LP-U-net compared to manual segmentation of MRI-based 3D models serving as gold standard is shown.

| Metrics | Acetabular models | Femoral models |
| --- | --- | --- |
| Dice Overlap Coefficients (%) | 95.8±2.1 (92.4–98.2) | 98.2±0.7 (96.4–98.8) |
| Average Surface Distance (mm) | 0.54±0.88 (0.1-2.98) | 0.15±0.06 (0.08-0.30) |
| Hausdorff Distance (mm) | 12.2±10.8 (3.2-38.6) | 4.48±1.39 (2.34-6.65) |

**References**

[58] Zeng, Guodong, et al. "3D U-net with multi-level deep supervision: fully automatic segmentation of proximal femur in 3D MR images." International workshop on machine learning in medical imaging. Springer, Cham, 2017.

[59] Schmaranzer, Florian, et al. "Automatic MRI-based three-dimensional models of hip cartilage provide improved morphologic and biochemical analysis." Clinical Orthopaedics and Related Research® 477.5 (2019): 1036-1052.

[60] Milletari, Fausto, Nassir Navab, and Seyed-Ahmad Ahmadi. "V-net: Fully convolutional neural networks for volumetric medical image segmentation." 2016 fourth international conference on 3D vision (3DV). IEEE, 2016.

[61] Zeng, Guodong, and Guoyan Zheng. "Holistic decomposition convolution for effective semantic segmentation of medical volume images." Medical image analysis 57 (2019): 149-164.
