# Supplementary material for: MRI-based 3D models of the hip joint enables radiation-free computer-assisted planning of periacetabular osteotomy for treatment of hip dysplasia using deep learning for automatic segmentation
Source: Eur J Radiol Open. 2020 Dec 18;8:100303. doi: 10.1016/j.ejro.2020.100303 (PMC7753932; doi:10.1016/j.ejro.2020.100303)
Supplement: Supplementary file 2 [file mmc2.docx]

**Video 1.** A video of a MRI-based 3D model of a symptomatic patient with pincer-type FAI due to acetabular retroversion that underwent simulation of an anteverting periacetabular osteotomy (PAO) is shown. The range of motion was simulated in 90° of flexion. Gradually the amount of internal rotation in 90° of flexion was increased to determine location of osseous impingement. The zone encircled by the red points signifies the impingement area.
